# Supplementary material for: Polyketide synthase gene clusters in the ericoid mycorrhizal fungus Oidiodendron maius: genome-wide prediction, expression profiling, and functional characterization of knockout mutants
Source: Front Plant Sci. 2026 Sep 16;17:1924430. doi: 10.3389/fpls.2026.1924430 (PMC13623903; doi:10.3389/fpls.2026.1924430)
Supplement: Supplementary file 1 [file SupplementaryFile1.zip › Supplementary Materials/Supplementary Folder 1.zip!/Oidma1_AssemblyScaffolds_Repeatmasked/knownclusterblast/cluster20/jgi.p_Oidma1_95066_mibig_hits.html]

| MIBiG Protein | Description | MIBiG Cluster | MiBiG Product | % ID | % Coverage | BLAST Score | E-value |
| --- | --- | --- | --- | --- | --- | --- | --- |
| CAJ96465.1 | Lysine/ornithine\_N-monooxygenase | BGC0000330 | Cupriachelin\_biosynthetic\_gene\_cluster | 41 | 100.451467269 | 291 | 1.8e-78 |
| AAY93340.1 | L-ornithine\_5-monooxygenase\_PvdA | BGC0000413 | Pyoverdine\_biosynthetic\_gene\_cluster | 38 | 100.451467269 | 285 | 1e-76 |
| ADJ63845.1 | L-ornithine\_N5-monooxygenase\_protein | BGC0000424 | Serobactins\_biosynthetic\_gene\_cluster | 40 | 98.6455981941 | 284 | 2.9e-76 |
| ACY06283.1 | L-lysine\_6-monooxygenase | BGC0001042 | Sanglifehrin\_A\_biosynthetic\_gene\_cluster | 38 | 98.8713318284 | 267 | 2.8e-71 |
| CAQ71838.1 | L-ornithine\_5-monooxygenase(L-ornithine\_N5-oxygenase)\_L-lysine\_6-monooxygenase\_(Lysine\_N(6)-hydroxylase);\_Siderophore\_biosynthesis\_protein | BGC0001189 | Taiwachelin\_biosynthetic\_gene\_cluster | 38 | 98.6455981941 | 267 | 2.8e-71 |
| CAM02311.1 | putative\_peptide\_monooxygenase | BGC0000349 | Erythrochelin\_biosynthetic\_gene\_cluster | 39 | 98.8713318284 | 266 | 4.8e-71 |
| AAZ55902.1 | putative\_peptide\_monooxygenase | BGC0000359 | Fuscachelin\_biosynthetic\_gene\_cluster | 39 | 97.7426636569 | 264 | 3.1e-70 |
| CBG75497.1 | putative\_peptide\_N-oxygenase | BGC0000423 | Scabichelin\_biosynthetic\_gene\_cluster | 38 | 100.902934537 | 254 | 1.9e-67 |
| AGE11900.1 | L-ornithine\_5-monooxygenase | BGC0000366 | Gobichelin\_biosynthetic\_gene\_cluster | 38 | 98.1941309255 | 254 | 2.5e-67 |
| CBZ42147.1 | peptide\_monooxygenase | BGC0001117 | Himastatin\_biosynthetic\_gene\_cluster | 37 | 98.4198645598 | 250 | 2.8e-66 |
| AMK48246.1 | L-ornithine-5-monooxygenase | BGC0001351 | Cahuitamycins\_biosynthetic\_gene\_cluster | 37 | 101.354401806 | 243 | 4.4e-64 |
| EFL06875.1 | peptide\_monooxygenase | BGC0000300 | Amychelin\_biosynthetic\_gene\_cluster | 37 | 100.225733634 | 242 | 7.5e-64 |
| WP\_039794392.1 | L-lysine\_6-monooxygenase | BGC0001211 | Albachelin\_biosynthetic\_gene\_cluster | 35 | 98.1941309255 | 240 | 3.7e-63 |
| CAB53328.1 | putative\_peptide\_monooxygenase | BGC0000325 | Coelichelin\_biosynthetic\_gene\_cluster | 34 | 101.58013544 | 239 | 8.3e-63 |
| ABV56589.1 | KtzI | BGC0000378 | Kutznerides\_biosynthetic\_gene\_cluster | 36 | 98.1941309255 | 228 | 1.9e-59 |
| BAM09327.1 | putative\_acinetoferrin\_biosynthesis\_protein | BGC0000295 | Acinetoferrin\_biosynthetic\_gene\_cluster | 24 | 101.128668172 | 129 | 9.2e-30 |
